# Supplementary material for: Association of parathyroid hormone and vitamin D with untreated hypertension: Is it different in white-coat or sustained hypertension?
Source: PLoS One. 2017 Nov 27;12(11):e0188669. doi: 10.1371/journal.pone.0188669 (PMC5703558; doi:10.1371/journal.pone.0188669)
Supplement: S1 File — (DOCX) [file pone.0188669.s001.docx]

Relationship between serum parathyroid hormone and vitamin D levels with untreated hypertension: Is it different in white-coat or sustained hypertension?

Abstract

Background:

Previous reports about the relationship between a high parathyroid hormone (PTH) and low vitamin D levels with blood pressure in different hypertension groups are conflicting.

Objective:

We studied serum PTH and vitamin D levels in white-coat (WCHT) and sustained hypertension (SHT) patients who had not been prescribed antihypertensive treatment. We also investigated the association between serum PTH and vitamin D levels with respect to blood pressure in SHT and WCHT patients.

Methods:

We included 52 SHT patients (54.06 ± 9.2 years, 32 newly diagnosed and 20 previously diagnosed with SHT who had not been treated with antihypertensive medication for 3 months or more), 48 WCHT patients (53.64 ± 9.5 years), and 50 normotensive (NT) healthy controls (53.44 ± 8.4 years) in our study. In addition to routine tests, PTH and vitamin D levels were measured.

Results:

Serum PTH levels were significantly higher in SHT patients not taking antihypertensive medications than in WCHT patients and NT controls (p=0.003). Although PTH levels were higher in WCHT than in NT groups, the difference was not statistically significant. In SHT patients, PTH levels showed a positive correlation with office systolic (r=0.363, p=0.008), office diastolic (r=0.282, p=0.038), home systolic (r=0.390, p=0.004), and home diastolic blood pressures (r=0.397, p=0.003). Serum vitamin D levels were similar in SHT, WCHT and NT groups (p=0.131). Vitamin D levels were not associated with blood pressures in either the hypertensive or control groups. Furthermore, no significant relation was found between vitamin D and PTH levels in SHT and WCHT groups.

Conclusion:

PTH levels were significantly higher in patients with SHT who are not on antihypertensive treatments as compared with WCHT patients and NT subjects. However, vitamin D levels were similar in SHT, WCHT and NT groups. Moreover, there is no relationship between PTH and vitamin D levels in either hypertensive or NT patients. Therefore, the measurement of PTH levels may be useful to differentiate between untreated SHT and WCHT patients, and suggests that elevated PTH has a role in the increase of blood pressure in SHT.

.

Key words: Hypertension, sustained, white-coat, untreated, parathyroid, vitamin D

INTRODUCTION

It has been reported that vitamin D and parathyroid hormone (PTH), aside from calcium and phosphorus metabolism, play important roles in the development of hypertension (HT) [1-5]. Previous studies have stated that lower levels of vitamin D are associated with incident HT [1,2]. Moreover, other studies have shown that elevated serum PTH levels are related with an increased risk of HT development [3,4] even in patients with normal serum PTH levels [5]. It is well known that primary hyperparathyroidism is associated with established HT and that parathyroidectomy significantly reduces blood pressure (BP) [6,7]. In addition, the association between BP and serum vitamin D and PTH levels has been widely studied [8-11]. While some authors reported an association between BP, serum vitamin D and PTH levels [8, 9], others did not find any relation [10,11]. Almost all of these investigations were conducted on HT patients who were receiving antihypertensive treatment [8-11]. One study showed that use of furosemide and dihydropyridine calcium-channel blockers was associated with higher PTH levels [12]. Another study reported that the intravenous infusion of the selective beta-1 adrenergic blocker esmolol increased serum PTH levels in healthy adults [13]. On the other hand, the use of thiazide diuretics or renin angiotensin aldosterone system inhibitors (angiotensin-converting enzyme inhibitors or angiotensin receptor blockers) was associated with lower PTH levels [12,14]. Conducting studies in HT patients who are not taking antihypertensive medications could attenuate the conflicts between investigations and reveal the real association between BP, vitamin D and PTH levels. Therefore, the objective of our study was to investigate serum vitamin D and PTH levels in patients with white-coat hypertension (WCHT) and sustained hypertension (SHT) who had not received antihypertensive treatment, thereby elucidating the possible correlation between BP, vitamin D and PTH levels in these patients.

MATERIALS AND METHODS

*Study Population*

The current study was conducted at our outpatient clinics (İskenderun Heart Center, Hatay, Turkey; Bülent Ecevit University, Department of Cardiology, Zonguldak, Turkey, and Sanko University, Department of Cardiology, Gaziantep, Turkey). We selected 52 SHT patients (54.06 ± 9.2 years, 32 with newly and 20 with previously diagnosesd SHT who had not been prescribed antihypertensive treatment for 3 months or more), 48 WCHT patients (53.64 ± 9.5 years), and 50 normotensive (NT) healthy controls (53.44 ± 8.4 years) among patients who had been referred to our clinics.

Exclusion criteria included the following: secondary HT, coronary artery or cerebrovascular disease, moderate or severe valvular heart disease, congenital heart disease, heart failure, diabetes mellitus, renal or hepatic dysfunction, systemic inflammatory disease, hematological system disorder, cancer, bone disorder, hyperthyroidism, obstructive sleep apnea, chronic obstructive pulmonary disease, pregnancy, previous gastrectomy or evidence of intestinal malabsorption, use of medications having an effect on vitamin D or PTH levels, and use of antihypertensive or antidepressant drugs.

The study was performed according to the Declaration of Helsinki and was approved by Sanko University Ethics Committee for Clinical Investigations, Gaziantep, Turkey.Written informed consent was obtained from all participants.

All patients and controls underwent full history taking, complete physical examination, and routine biochemical blood analyses. Transthoracic echocardiography and 12-lead electrocardiography were performed in all patients and controls. Height (in meters) and body weight (in kilograms) were measured to calculate body mass index (kg/m^2^).

*Measurement of Office and Home Blood Pressure*

Office BP was measured in a quiet environment with a mercury sphygmomanometer after 5 min of rest, from both upper limbs, while subjects were seated. Systolic (SBP) and diastolic blood pressures (DBP) were defined with Korotkoff phase I and V sounds, respectively. The two BP measurements were obtained in 1-minute intervals, and the mean of the two measurements was calculated.

Home BP was measured for 5 days using an electronic BP measuring device (Microlife BP A90 device, model BP 3BTO-, Microlife AG, Switzerland), according to a standard procedure for which subjects were trained [15, 16]. Specifically, they were asked to measure their BP every morning (between 6 and 10 AM), three times at 1-min intervals, and every night (between 6 and 10 PM), three times at 1-min intervals while they were seated and after they had rested for more than 5 min. Moreover, subjects were instructed to record each measurement taken by the device on a form provided by the attendants. After this 7-day period, patients returned their devices and the written BP results. The measurements taken on the first day were excluded.

Patients with mean office SBP ≥140 mm Hg and/or DBP ≥90 mm Hg were considered to be hypertensive. Hypertensive patients with mean home BP measurement ≥135/85 mmHg were diagnosed as having SHT, while patients with mean home BP measurement <135/85 mmHg were considered as having WCHT [15,16].

*Laboratory Tests*

Venous blood samples were obtained during the morning (8– 10 AM) after 12 hours of fasting. Serum biochemical and hematologic tests were performed by standard methods. Serum vitamin D levels were measured using high-performance liquid chromatograph mass spectrometry (LC-MS/MS Shimadzu 8040, Kyoto, Japan). Serum PTH measurements were taken by using electrochemiluminescence on the E 170 Modular Analytic System (Roche, Sweden).

*Statistical Analysis*

Statistical analyses were performed by using SPSS (Statistical Package for the Social Sciences ver. 16, SPSS Inc., Chicago, Illinois, USA) software. Continuous variables were defined as mean ± standard deviation; categorical variables were presented as percentage values. The Kolmogorov–Smirnov test was used for the analysis of normal distribution of variables, and the values that did not distribute normally were log-transformed. Analysis of variation (ANOVA) tests were performed for the comparison of continuous variables among the groups. The Tukey test was used in post-hoc analysis for variables that were found to differ. The chi-square test was used to compare categorical variables between the groups. The relation between numerical variables was identified by using Pearson’s correlation test. Multiple linear regression analysis was used to assess the independent association of BP with clinical and laboratory parameters. A two-sided p value less than 0.05 was considered statistically significant.

RESULTS

Participant demographic characteristics and BP measurements are given in Table 1. Age, sex distribution, body mass index, and smoking rate did not differ among the study groups. The office and home BPs of patients with SHT were significantly higher than those subjects with WCHT and NT (Table 1). Although the office BP measurements were significantly higher in WCHT patients than in NT subjects, the home BP measurements were comparable between these two groups.

The laboratory parameters of the study groups are given in Table 2. The levels of basic laboratory parameters were similar in SHT, WCHT and NT groups. Vitamin D levels were comparable in SHT, WCHT and NT groups (19.55 ± 7.8, 21.73 ± 8.4, 22.92 ± 9.4 ng/mL, respectively, p=0.131) (Table 2, Figure 1). However, log-transformed PTH levels were higher in patients with SHT as compared with WCHT and NT subjects (p=0.003) (Table 2, Figure 2).

Bivariate correlation analysis showed that, in patients with SHT, PTH levels did not correlate with age, body mass index, calcium, phosphorus, thyroid-stimulating hormone or vitamin D (Table 3). However, PTH levels were positively correlated with office SBP (r=0.363, p=0.008), office DBP (r=0.282, p=0.038), home SBP (r=0.390, p=0.004), and home DBP (r=0.397, p=0.003; Table 3, Figure 3). In WCHT patients, PTH levels did not correlate with laboratory parameters, or office or home BP measurements (Table 3).

Multiple linear regression analysis revealed that PTH levels were the only independent correlate between home SBP and DBP in the entire study group (home SBP, β=0.32, p=0.001; home DBP, β=0.35, p<0.001, Table 4).

DISCUSSION

In our study, PTH levels were significantly higher in patients with SHT than in WCHT patients and NT healthy subjects. We found a positive correlation between PTH levels and BP in SHT patients. However, we did not find a significant difference in vitamin D levels between NT subjects and SHT and WCHT patients. Additionally, we did not find an association between PTH and vitamin D levels in SHT and WCHT groups.

*Vitamin D and Hypertension*

Vitamin D is a hormone that plays an important role in regulation of calcium and phosphorus metabolism. Recent studies have shown that vitamin D is associated with many diseases, including HT, cardiovascular diseases, diabetes mellitus and metabolic syndrome [2,16,17]. Many authors have stated that a decrease in vitamin D levels increases the risk of HT [1-2]. However, results of studies that investigated the relationship between vitamin D levels and BP in HT patients are conflicting [8-11]. Some studies have reported higher levels of vitamin D in HT patients as compared with NT subjects [8,9]. In these studies, an inverse association between vitamin D levels and BP was found [8,9]. On the other hand, a number of cross-sectional and prospective studies stated that there was no relation between BP and vitamin D levels [10,11,18,19]. Chan et al. reported that there was no significant association between BP and vitamin D levels in their cross-sectional study performed on 939 male patients aged 65 years and older [18]. Moreover, in a prospective study conducted by [Skaaby T](https://www.ncbi.nlm.nih.gov/pubmed/?term=Skaaby%20T%5BAuthor%5D&cauthor=true&cauthor_uid=22986625) et al., a significant relation was not established between vitamin D levels and BP of 6,784 individuals in a 5-year follow-up [19]. We found the absence of an association between vitamin D levels and BP in our study group, including both hypertensive patients and NT subjects⎯results that are consistent with those of the studies conducted by Chan et al. and [Skaaby T](https://www.ncbi.nlm.nih.gov/pubmed/?term=Skaaby%20T%5BAuthor%5D&cauthor=true&cauthor_uid=22986625) et al. [18,19].

It has been reported that approximately 15% to 30% of patients with increased in-clinic BP readings have WCHT and about 52 % of these develop SHT within 11 years of follow up [20-22]. Some authors have considered WCHT to be an innocent disease and reported that the risk of cardiovascular and cerebrovascular events were similar in WCHT and NT subjects [23,24]. However, other authors have stated that WCHT is not a benign disease. They reported that target organ damage, including left ventricular hypertrophy and systemic atherosclerosis, were higher in WCHT than NT subjects [25,26]. Furthermore, WCHT has been reported as increasing the risk of cardiovascular mortality [27].

Vitamin D levels in WCHT have not been well studied. To our knowledge only one study in the literature investigated vitamin D levels in WCHT [8]. In that study, Alpsoy et al. reported that the vitamin D levels were similar in WCHT and NT subjects (34.3 ± 3.6 and 36 ± 5 ng/mL, respectively) [8]. Similarly, we did not find significant differences in vitamin D levels between WCHT and NT patients.

*Parathyroid horormone levels and Hypertension*

PTH regulates calcium, phosphorus and magnesium levels in the bones and blood through the [kidneys](http://www.yourhormones.info/glands/kidneys/), bones and intestines. An increase in serum PTH levels leaves patients prone to develop incident HT [3-5]. The association between PTH and BP in HT patients has been studied by many researchers, nevertheless the results of their studies are conflicting [8-10,28]. Some investigators have stated that HT patients had higher PTH levels compared with NT subjects and reported the highest PTH values in patients with stage II HT. [9,28]. They also found a positive correlation between PTH levels and BP in these patients [9,28]. However, other investigators did not find any relation between PTH levels and BP and reported similar PTH levels in SHT and NT subjects [8,10].

Differences in sex, age, and race between subjects included in the aforementioned studies may be responsible for the discrepancies between their results. We suggest that the use of antihypertensive drugs plays an important role in these inconsistencies. Antihypertensive drugs decrease both systolic and diastolic BP, thus they may attenuate the significance of an association between BP and PTH levels. Furthermore, some antihypertensive drugs themselves affect PTH levels [13,29]. [Schmitt CP](https://www.ncbi.nlm.nih.gov/pubmed/?term=Schmitt%20CP%5BAuthor%5D&cauthor=true&cauthor_uid=14638923) et al. reported that the beta blocker esmolol increases serum PTH levels [13]. [Zaheer S](https://www.ncbi.nlm.nih.gov/pubmed/?term=Zaheer%20S%5BAuthor%5D&cauthor=true&cauthor_uid=26748479) et al. showed that dihydropyridine calcium-channel blocker use is associated with significantly higher PTH levels in HT subjects [12]. However, other authors have reported that the use of angiotensin-converting enzyme inhibitors, angiotensin receptor blockers, or spironolactone are associated with lower PTH levels [14,29,30].

Only a few studies in the literature have investigated the association between PTH levels and BP in HT patients who did not use antihypertensive treatment [14,31]. Kim et al. reported a positive correlation between serum PTH concentrations and SBP and DBP in subjects not taking antihypertensive medication [31]. Another study, conducted by Brown et al., revealed that HT patients not taking anti-hypertensive medication had higher PTH levels than did NT patients [14]. In our study, we did not include patients who were receiving antihypertensive treatment. The patients included were either newly or previously diagnosed with HT who had not been prescribed antihypertensive treatments. In accordance with previous studies, we found a positive association between serum PTH levels and BP in HT patients, i.e., SBP and DBP increased with an increase in PTH levels.

There are multiple mechanisms explaining how PTH may increase BP and cause HT development. PTH activates the renin-angiotensin-aldosterone system, resulting in renin release, and thus, increases BP [32]. PTH itself impairs endothelial vasodilatory function and causes arterial vessel thickening through its prosclerotic effect on smooth muscle cells that results in development of HT [33,34]. PTH also causes renal damage and renal calculi, after which HT may develop [35].

Although vitamin D and PTH are interrelated in many physiological pathways of the body, we did not find any association between vitamin D and PTH in our HT patients. This condition can be explained by factors other than vitamin D levels that might influence PTH levels [36-38]. It has been reported that consuming high phosphorus and low calcium diets elevates PTH secretion [36]. Moreover, in vitro studies have demonstrated that high phosphate administration induces PTH release and a physiologically high dose of caffeine concentration inhibits PTH secretion [37-38].

Regarding WCHT, we found only one study in the literature that investigated PTH levels in patients with this condition [8]. Alpsoy et al. did not find significant differences in PTH levels between WCHT patients and NT subjects (42, 30–55, and 41, 30–55 pg/mL, respectively) [8]. In our study, in accordance with Alpsoy et al., we found similar PTH levels in WCHT and NT subjects.

*Limitations*

Our study has some limitations; first, the sample size of our study is small. Second, the cross-sectional design of the study does not permit us to assume cause and effect relationships. Third, we did not collect data about sunlight exposure and the amount of dietary intake of caffeine, phosphorus, calcium and vitamin D⎯factors that might affect vitamin D and PTH levels. Fourth, because of wide-range variations in the serum vitamin D and PTH levels of our patients, the use of single measurements of serum vitamin D and PTH may have minimized the significance of the association between these parameters and BP.

In conclusion, this study shows that Vitamin D levels are similar among SHT, WCHT and NT patients. However, PTH levels are significantly higher in patients with SHT who are not on antihypertensive treatments when compared with WCHT patients and NT subjects. PTH levels in SHT patients are positively associated with SBP and DBP, supporting the possibility that PTH plays a role in the increased BP in these patients. Importantly, more comprehensive studies are essential to confirm our findings and explore the importance of PTH in HT, especially in untreated SHT patients.

Acknowledgments

We thank Özel Yaşamlab Tıbbi Laboratuvarı for its work on the present research.
